# Exploration of Allergic Rhinitis: Epidemiology, Predisposing Factors, Clinical Manifestations, Laboratory Characteristics, and Emerging Pathogenic Mechanisms

**DOI:** 10.7759/cureus.71409

**Published:** 2024-10-14

**Authors:** Che Othman Siti Sarah, Noor Suryani Mohd Ashari

**Affiliations:** 1 Department of Immunology, School of Medical Sciences, Universiti Sains Malaysia, Kubang Kerian, MYS

**Keywords:** allergic rhinitis, clinical manifestation, laboratory diagnosis, pathophysiology, tight junction

## Abstract

Allergic rhinitis (AR) is a widespread allergic condition, with its prevalence continuing to rise globally. This disease has a significant impact on patients' quality of life. Understanding the underlying pathophysiology is important to develop better-targeted therapies. For decades, the primary assumption has been that an allergy is caused by unbalanced and overactive immunological responses against allergens, driven mainly by activated T helper 2 (Th2) cells and due to aberrant T-regulatory cells. The more recent hypothesis that is gaining attention relies on the dysregulation of the epithelial barrier, which might result in allergen uptake as a primary defect in the pathogenesis of allergic reactions. The nasal epithelial barrier is considered a crucial first line of defense in the upper airway, as it protects the host’s immune system from exposure to allergens. Thus, this review will discuss AR’s epidemiology, predisposing factors, clinical manifestations, laboratory characteristics, and pathogenic mechanisms.

## Introduction and background

Allergic rhinitis (AR) is an IgE-mediated inflammatory disorder of the nasal mucosa, resulting from immune system dysregulation caused by the infiltration of allergen and cytokine production imbalance [1-5]. According to several studies [6-8], AR can have various detrimental effects on a person’s quality of life, affecting mood and daily activities, lowering scholastic performance, limiting social interaction, and increasing financial costs.

Survey methodology

This review focuses on AR, its clinical and laboratory characteristics, and its pathophysiological mechanisms. All articles were searched and reviewed by two researchers (NSMA and COSS) using electronic databases such as PubMed and Google Scholar. The references mentioned in this review were obtained from databases up to 2023. The following keywords were used in this review: allergic rhinitis, epidemiology, predisposing factors, diagnostic criteria, pathophysiology, epithelial barrier, and tight junction.

## Review

Epidemiology of AR

AR is the most common of all allergic diseases, affecting around 10%-40% of the population globally. It has been estimated that 25% of children and 40% of adults worldwide suffer from AR [9]. AR prevalence in the Western population is estimated to be about 30% [5]. In the United States, the prevalence of AR varies from 9% to 42% [10] and is the third most common chronic disease among children and adolescents [11]. In the United Kingdom, the prevalence reaches 26% in adults, with a peak in the third and fourth decades of life [12,13].

Across the African continent, research indicates significant prevalence rates, with up to 20% of young people in South Africa affected and 9.1% in Ghana [14-16]. AR also affects a substantial population in Asia, ranging from 27% in South Korea [17] to 32% in the United Arab Emirates [18]. In addition, a survey conducted across the Asia-Pacific regions found that 2.5% of the population in the Philippines and 13.2% in Australia had a physician diagnosis of AR [19]. In Malaysia, the prevalence of AR is 7.1% in adults, 11.2% among primary school students aged 7 to 12, and 31.7% to 55.5% in junior high school students [19,20]. There are variations between nations, perhaps due to variations in immune responses and allergens.

Predisposing factors of AR

AR is commonly found in all age groups, from early childhood to adolescence to late adulthood [9,21,22]. AR’s prevalence was found to peak between the ages of 16 and 24, and it significantly declined in both men and women in the 65-84 age group [23-26]. According to a systematic review and meta-analysis, AR’s prevalence in adults was not sex-specific [27]. However, in childhood, there is a male predominance, but after age 15, females are more affected [28-30]. Female students had a 42.8% higher chance of having AR than male students, according to survey results among university students in Turkey with a mean age of 20.71 ± 3.12 years [31].

Exposure to cigarette smoke reportedly can worsen allergic airway inflammation [32]. According to previous studies, cigarette smoke directly damages the epithelial cells and results in increased permeability, the release of cytokines and chemokines, and shifted lymphocyte balance toward T helper 2 (Th2) cells [33]. Additionally, cigarette smoke exacerbates nasal allergic reactions while increasing the production of IL-5 and the level of serum IgE [34-36].

The increasing prevalence of allergies has been associated with the increased prevalence of obesity [37-39]. Studies demonstrated that leptin, which is an adipokine and a fat-related hormone, was associated with allergen exposure and subsequently associated with AR’s severity [40,41].

Clinical manifestations of AR

AR is closely associated with other allergic diseases, such as asthma and atopic dermatitis, due to shared immune dysregulation, particularly IgE-mediated inflammation [42]. Exposure of the nasal mucosa to allergens results in the production of IgE, activation of eosinophils, and degranulation of mast cells and basophils [43]. These events lead to the release of histamine, leukotrienes, and prostaglandins, which subsequently result in the production of clinical manifestations [44]. Sneezing, watery anterior rhinorrhea, nasal itching, nasal obstruction, and postnasal drip are the most common clinical manifestations [44-46]. Additionally, AR patients frequently experience non-nasal manifestations, including chronic cough; persistent headache; eye, ear, and throat symptoms; and cognitive impairment [9,47].

Based on the allergen that triggers the symptoms, AR is typically classified as either seasonal (SAR) or perennial (PAR) [48,49]. Animal dander or house dust mites (HDMs) (*Dermatophagoides pteronyssinus, D. farinae,* and *Blomia tropicalis*) are the most frequent causes of PAR, which affects patients year-round, while pollen allergens are the primary cause of SAR, affecting patients during specific seasons [3,50].

Laboratory characteristics and diagnostic criteria of AR

Typically, the presence of total and specific IgE in the serum supports the diagnosis of AR. Previous studies have shown that AR patients have higher total IgE levels than nonallergic individuals [51,52]. Multiple tests, including in vivo skin tests such as the skin prick test (SPT) and intradermal test (IDT) and in vitro serum specific immunoglobulin E (ssIgE) assays, can be used to identify the specific allergens that cause the development of IgE antibodies in AR [53,54]. SPT and ssIgE assays are the most common diagnostic approaches. Both tests have high specificity and sensitivity in determining the sensitization to common allergens [53,55].

SPT is considered the primary method to diagnose sensitization in AR [3]. It is the most frequently used and effective test for ssIgE antibody detection due to being rapid, simple, and affordable [54,56]. The test is performed by introducing specific allergens into the skin of allergic individuals using a lancet. Dermal mast cells degranulate as a result of the cross-linking of allergen-specific IgE bound to their membrane receptors [54], which results in the rapid release of histamine and other mediators. This induces an immune response that is clinically characterized by a wheal and flare that can be measured to determine the degree of cutaneous sensitization [54].

In vitro laboratory testing for ssIgE antibodies is performed using commercially available test panels [56,57]. The ssIgE test can identify the presence of IgE antibodies that bind to molecular elements or allergens [54]. Despite being more expensive than SPT, the ssIgE test is beneficial for patients when skin testing is unavailable or impossible to perform due to extensive skin diseases or other causes that can complicate skin testing [58,59]. In addition, the ssIgE test is not affected by antihistamine drugs, which is useful for patients who are unable to stop the medications [54].

Other tests to consider include the IDT test, which can also detect IgE-mediated allergies [54]. In this test, a minute amount of allergen is injected intradermally with a needle to form a small bleb. The outcome measured is an increase in the size of the wheal-and-flare reactions after 20 minutes [54,60]. Additionally, if both in vitro and in vivo tests are negative or inconclusive, a basophil activation test can be performed. Basophils have been recognized as important effector cells in immediate hypersensitivity reactions [54]. The degranulation of basophils can be detected and quantified by the flow cytometry technique [54,61].

A definitive diagnosis of AR is made based on symptoms such as sneezing, nasal itching, and watery rhinorrhea; physical findings, including edematous intranasal mucosa; and identification of specific causative allergens based on in vivo skin tests such as SPT or in vitro ssIgE assays [62], as recommended by the Allergic Rhinitis and its Impact on Asthma (ARIA) guidelines [9].

Pathophysiologic mechanism of AR

Early and Late Phase of AR

AR’s pathogenesis is complex, consisting of early- and late-phase allergic reactions. The early phase begins with allergen sensitization. Soluble allergens from inhaled pollen and other aeroallergens are rapidly eluted upon contact with the mucous membranes of the nasal mucosa, which induces the production of IgE antibodies and triggers the humoral response [63,64]. The invaded allergens are processed by antigen-presenting cells (APCs), such as dendritic cells (DCs) at a mucosal site, activating naive CD4+ T cells to become Th2 cells [64,65]. This induces Th2 cells to secrete cytokines such as IL-4 and IL-13 [64,65]. This process converts the B cells into allergen-specific IgE-producing plasma cells, which then produce IgE antibodies [65]. IgE antibodies will subsequently bind to mast cells and basophils [64]. Degranulation of mast cells and basophils occurs upon re-exposure to the same allergen. This process is initiated by cross-linking of specific IgE antibodies on FceR1 by their relevant allergen [64-66].

In this early phase, the interaction triggers a cascade of reactions in which mast cells and basophils release mediators, such as histamine, chemokines, cytokines, and adhesion molecules, leading to increased leukocyte production in the bone marrow [65,66]. The rapid onset of acute nasal symptoms like nasal congestion, rhinorrhea, or itching are produced by the substances that act on the vessels and glands of the nose [64].

**Figure 1:**
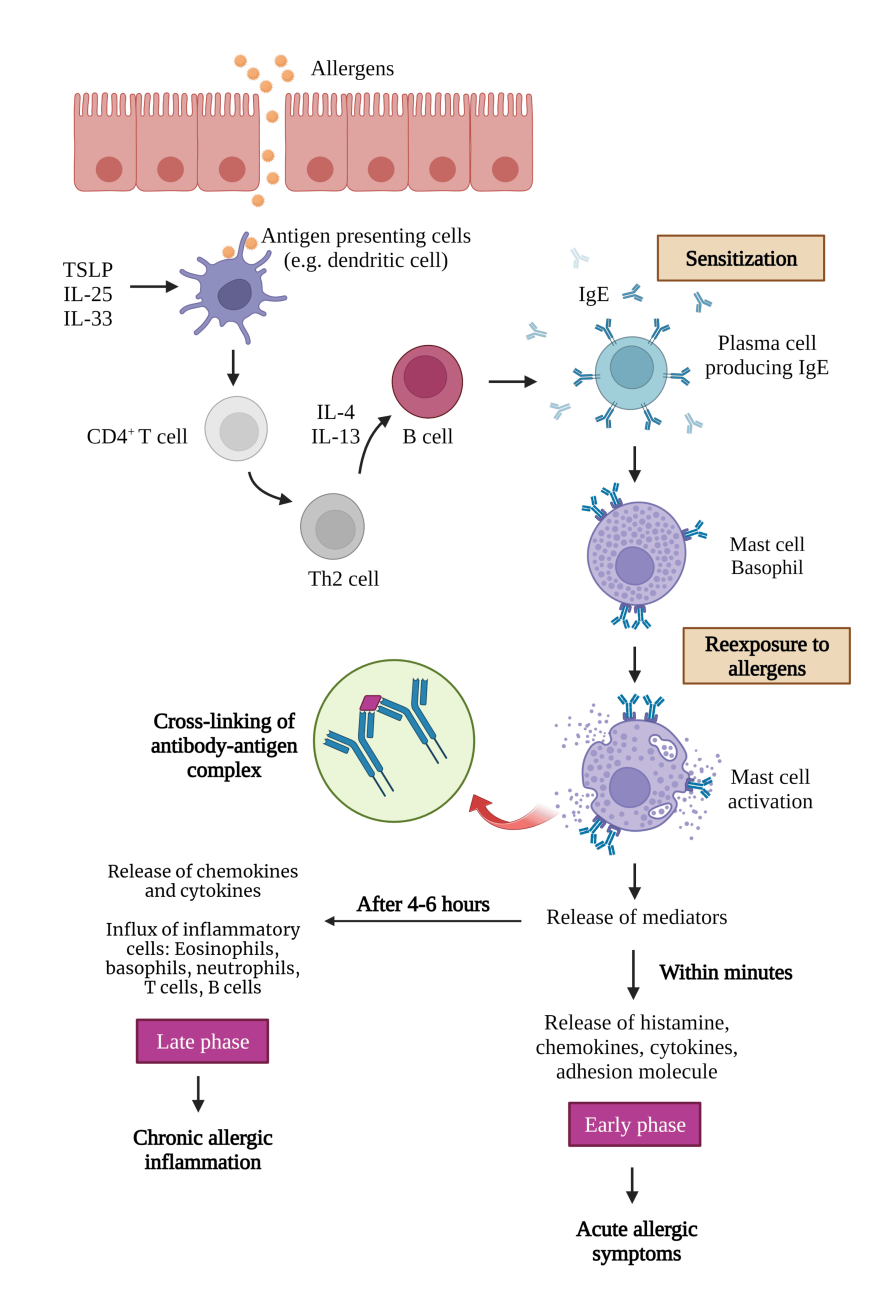
The pathophysiology of allergic rhinitis TSLP: thymic stromal lymphopoietin; IL: interleukin; IgE: immunoglobulin E; Th2 cell: type 2 helper T cell Image created with BioRender.com.

The early-phase allergic reaction is usually followed by the late-phase response, which occurs four to six hours after antigen stimulation. The late-phase response is characterized by prolonged symptoms such as sneezing, rhinorrhea, and sustained nasal congestion lasting 8-24 hours. Local Th2 lymphocyte activation by DCs leads to the release of chemokines and cytokines that orchestrate the influx of inflammatory cells, such as eosinophils, basophils, neutrophils, T cells, and B cells, to the mucosa and increase the number of allergen targets [65]. The mucosa becomes more sensitive to allergens and environmental irritants. In addition, allergen exposure further stimulates IgE production [65]. In a series of time-dependent phases, these effector cells, mediators, and cell signaling molecules work in a complex network of interactions that result in specific symptoms and the inflammatory morphology of AR [66]. AR’s pathophysiology is presented in Figure 1.

Nasal Epithelial Barrier Disruption

The nasal epithelial barrier plays a vital role in both innate and adaptive mucosal immunity through the activation of functional molecules such as pro-inflammatory cytokines, chemokines, and growth factors [67]. This epithelial barrier seals the nasal passage and underlying tissue to prevent the entry of pathogens, allergens, and other foreign particles, as well as restrict the intercellular passage of fluid by connecting the epithelial cells [46,68].

**Figure 2:**
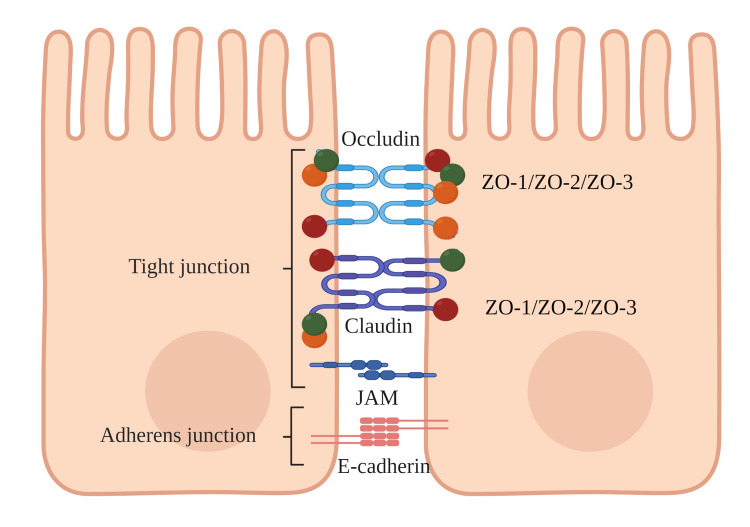
The interaction of TJs with either one of the ZO proteins at the epithelial cells. TJs: tight junctions; ZO: zonula occludens Image created with BioRender.com.

The physical barrier mainly comprises different junctional complexes that connect the epithelial cells [69]. Tight junctions (TJs) are multiprotein complexes located on the most apical side of epithelial cells. They comprise transmembrane proteins like claudins, occludin, junctional adhesion molecules, and intracellular proteins such as ZO-1, ZO-2, ZO-3, and tricellulin. Additionally, C-interacting proteins seal off the paracellular space between the epithelial cells [46,69]. Adherens junctions (AJs) are a form of cell-cell adhesion structures, constituted of E-cadherin and the cytoplasmic p120-, β-, and α-catenin [69,70]. AJs form apical junctional complexes with TJs, which control the epithelial cell-to-cell contact, actin cytoskeleton regulation, intracellular signaling pathways, and transcriptional regulation [69]. Desmosomes are found beneath the apical junctional complexes, which are specialized for strong adhesion and responsible for the mechanical stability between adjacent cells [69,71]. These cell junctions collaborate to create the intracellular connection between the cells to limit the passage of foreign molecules and protect the underlying tissue from exposure to harmful and allergenic stimuli [46,68]. The structure of the epithelial barrier is illustrated in Figure 2.

Dysfunction of these epithelial barriers may contribute to allergic disease by increasing exposure of the underlying tissue to environmental allergens [46]. Epithelial barrier disruption has been linked to various human diseases such as inflammatory bowel disease, celiac disease, and functional dyspepsia [72-74]. In allergic diseases, the disruption is observed in the epithelial cells of patients with atopic dermatitis, asthma, chronic rhinosinusitis, and AR [46,75,76].

Screening studies using microarray gene expression, RNA sequencing, and nasal mucus proteomics have suggested barrier dysfunction’s role in AR [5,68,77,78]. Multiple studies have found decreased expression of epithelial cell junction protein in AR [5,68]. Immunohistochemical and immunoblotting studies reported a decrease in E-cadherin expression in the nasal epithelium of patients with AR [52]. Previous studies saw decreased ZO-1 and occludin expression as measured by reverse transcription-quantitative polymerase chain reactions and weak immunofluorescence staining in AR biopsy specimens [68,79]. As a result of the breakdown of TJs, inflammatory cells may flood into the lumen, causing tissue injury or inflammation [76].

There are two hypotheses regarding the destabilization of the TJs: direct proteolysis and disruption by inflammatory cytokines [46]. Invading allergens, such as HDM allergens, contain protease, which is capable of disrupting the TJ molecules [80-82]. Proteolytic activity can be explained by the action of HDM cysteine proteinase antigen *D. pteronyssinus* [80]. This HDM proteases can reduce Th1 polarization by mediating Th2-biased immune responses [83,84]. For example, *D. pteronyssinus*deconstructs the IL-2 receptor involved in Th1 proliferation to promote Th2 proliferation [83]. *D. pteronyssinus* has been reported to cleave extracellular domain sites in occludin and claudin-1, leading to the degradation of TJs in the epithelium. This results in increased epithelial permeability, thus increasing the accessibility of the dendritic APCs residing beneath the epithelial barrier [80]. Furthermore, *D. pteronyssinus* has also been shown to cause a time-dependent breakdown of TJ and ZO-1 mislocalization from TJ [85].

Furthermore, serum levels of IL-13 and IL-4, both of which are classical Th2 cytokines, are elevated in AR patients [86]. IL-13 and IL-4 stimulation increased the expression of transient receptor potential vanilloid 4 (TRPV4) channels in cultured normal epithelial cells [87]. TRVP4 is a calcium-permeable channel found in respiratory epithelial cells [87,88]. TRVP4 expression was found to be higher in AR patients compared to the healthy control group [87]. Der p 1 reduced the expression of ZO-1 and E-cadherin after 24-hour stimulation in the presence of the TRVP4 agonist GSK1016790A. This indicates that the increase of TRVP4 in AR patients may exacerbate the destruction of the epithelial barrier caused by HDM-mediated activation of TRPV4 [87].

IL-33 is another inflammatory cytokine that has been implicated in AR pathogenesis. It is constitutively expressed and localized in the nucleus of nasal epithelial cells. IL-33 is known to induce Th2 cytokine production in Th2 cells, eosinophils, and mast cells [89]. In Japan, increased IL-33 expression has been found in the serum of SAR patients, thus revealing the association between IL33 and AR [90]. Using the ragweed pollen-induced murine model, IL-33 was constitutively expressed in nasal epithelial cells and showed an accumulation of eosinophils and basophils [89]. In addition, the IL-33 level was increased in the sinus mucosa and significantly correlated with the total nasal symptom score in HDM-sensitized AR patients [91]. Intranasal administration of IL-33 showed decreased expression of occludin and ZO-1 in control mice [92].

## Conclusions

AR is highly prevalent globally. A deeper understanding of AR’s pathophysiology, including the roles of immune responses and environmental triggers, is essential for developing more effective treatment strategies. Insights into the involvement of the nasal epithelial barrier in AR pathogenesis highlight the importance of exploring barrier integrity as a potential therapeutic target. Future research focused on this aspect could lead to innovative and practical approaches to managing AR, ultimately improving outcomes and quality of life for affected patients.
